# Multi-Sensor Spatiotemporal Feature Fusion for Early Warning of Cable Fires in Power Cable Tunnels

**DOI:** 10.3390/s26165179

**Published:** 2026-08-16

**Authors:** Mingming Wang, Dong Li, Xiaoyun Sun, Haiqing Zheng

**Affiliations:** 1 Hebei Provincial Collaborative Innovation Center of Transportation Power Grid Intelligent Integration Technology and Equipment, Shijiazhuang Tiedao University, Shijiazhuang 050043, China; wangmm@stdu.edu.cn (M.W.); zhqneu@163.com (H.Z.); 2 School of Electrical and Electronic Engineering, Shijiazhuang Tiedao University, Shijiazhuang 050043, China; 1202409019@student.stdu.edu.cn; 3 School of Electronic and Control Engineering, North China Institute of Aerospace Engineering, Langfang 065000, China

**Keywords:** spatiotemporal fusion, cable fire early warning, multi-sensor data fusion, power cable tunnel, spatial relationship modeling among sensor nodes

## Abstract

Power cable tunnels are typical enclosed cable-routing spaces in which cable overheating, insulation aging, and partial discharge may gradually develop into fire hazards. During the early stage of cable fires, abnormal sensor responses are often weak, localized, and continuously evolving, which increases the difficulty of early warning based on a single sensor or a single temporal feature. Motivated by cable fire early warning in power cable tunnels, this study uses cable-fire records from a publicly available indoor EN 54 fire-test-room dataset with distributed multi-sensor nodes to evaluate the proposed model under controlled laboratory conditions. In monitoring scenarios with fixed sensor nodes, temporal-only modeling methods often struggle to simultaneously characterize short-term variations, temporal evolution, and spatial differences in node responses. To address this limitation, this study proposes a multi-sensor spatiotemporal feature fusion model that integrates a gated recurrent unit (GRU), a Modern Temporal Convolutional Network (ModernTCN), and an enhanced graph convolutional network (GCN+). The proposed model adopts the ModernTCN as the temporal modeling backbone. A GRU module is introduced at the front end to encode local fluctuations and short-term continuous changes between consecutive time steps, while GCN+ is embedded at the intermediate feature stage of the backbone to model spatial correlations and cross-node coordinated responses among fixed sensor nodes. Experimental results show that the proposed model achieves strong classification performance in the cable fire early warning discrimination task, with a test accuracy of 0.9884 and a false negative rate (FNR) reduced to 0.0150. The comparative experimental results indicate that the proposed model achieves better overall performance than typical temporal baseline models. The ablation study further shows that, under the experimental settings of this study, the introduction of GRU and GCN+ leads to overall improvements in the main evaluation metrics, suggesting that both modules provide a certain enhancement to the cable fire early warning discrimination performance of the ModernTCN backbone.

## 1. Introduction

With the continuous development of urban underground space and the ongoing construction of distribution networks, the scale of centralized cable routing in power cable tunnels has continued to expand. Potential hazards arising from long-term cable operation, including insulation aging, partial discharge, overheating, and joint faults, have gradually become important factors affecting the safety of power cable tunnels. Compared with ordinary indoor environments, power cable tunnels are typically characterized by a long and narrow spatial layout, a high degree of enclosure, limited access points, and constrained operation, maintenance, and emergency response conditions. Once cable-related hazards develop into a fire, hot smoke and toxic gases can readily accumulate within the enclosed tunnel space and propagate along the tunnel corridor. Meanwhile, the fire may spread along the direction of cable routing, which substantially increases the difficulty of on-site response and may lead to circuit tripping, power supply interruptions, and damage to associated equipment. Existing accident analyses and experimental studies of cable fires in power cable tunnels have shown that cable insulation aging, partial discharge, and short-circuit faults can all trigger fires, and that such events may cause substantial economic losses and risks to power supply reliability [1,2,3,4]. Therefore, how to use multi-source monitoring information from the early stage of cable fires to perform early warning discrimination of fire risk is an important issue for improving the safe operation of power cable tunnels. In this study, the proposed model is developed for cable-fire early warning in power cable tunnels, and its feasibility is evaluated using cable-fire records selected from a publicly available indoor EN 54 fire-test-room dataset.

In fire monitoring and early warning, earlier studies mainly determined fire states based on sensor signals such as smoke, temperature, and gas concentration. Because threshold-based alarms from a single sensor are susceptible to environmental fluctuations, sensor noise, and non-fire disturbances, multi-source sensor information fusion has gradually become an important approach for improving alarm reliability. Khan et al. [5] systematically reviewed advances in fire sensing technologies from the perspectives of sensor type, detection mechanism, and application scenario, and noted that signals such as temperature, smoke, gas, and flame can serve as important monitoring cues for fire recognition. Fonollosa et al. [6] summarized advances in fire detection from the perspective of chemical sensor systems and their signal-processing algorithms, emphasizing the role of gas sensors and multivariate data processing methods in early fire detection and false-alarm mitigation. In terms of multi-sensor fusion-based alarms, Gottuk et al. [7] proposed a fire alarm algorithm based on multi-feature signals, and Chen et al. [8] verified the effectiveness of joint detection based on smoke and gas sensing information in fire recognition. Furthermore, Jing et al. [9] applied Bayesian networks to multi-sensor fire alarm systems, Ding et al. [10] implemented multi-source information fusion for building fires based on D-S evidence theory, and Rachman et al. [11] adopted fuzzy logic rules to process multi-sensor data in complex indoor scenarios. Overall, these methods can use multi-source information to mitigate the false-alarm problem in single-sensor alarms; however, their decision-making processes typically rely on manually defined rules, thresholds, prior probabilities, evidence assignments, or membership functions, and thus provide insufficient characterization of the continuous evolution of sensor responses during the early stage of fire development.

To enhance the ability of models to characterize nonlinear relationships in multi-source sensor data, machine learning and shallow neural network methods have been introduced into fire detection tasks. Andrew et al. [12] constructed an intelligent classifier for early-stage fires through multistage feature selection, and Wu et al. [13] used a backpropagation neural network (BPNN) to achieve indoor fire early warning based on multi-sensor information. Furthermore, Xiao et al. [14] combined a genetic algorithm-optimized BPNN, a particle swarm optimization-based least-squares support vector machine, and D-S evidence fusion to achieve high-sensitivity fire detection and early warning in intelligent building scenarios. Baek et al. [15] developed a real-time fire detection model based on support vector machines to characterize the time-series dynamics of multichannel sensor signals under different fire types; Jana et al. [16] adopted an ensemble learning method to improve the fire recognition performance of multimodal sensor data in smart buildings. Compared with traditional fusion methods based on rules or evidence combination, these methods can learn discriminative patterns for distinguishing fire and non-fire states from sample data, thereby reducing their dependence on fixed thresholds and predefined fusion rules. However, the modeling focus of these methods is mostly limited to feature selection, similarity measurement, or shallow classifier design. Although they can characterize certain nonlinear relationships, their ability to model trend evolution, short-term fluctuations, and short- and long-term temporal dependencies in continuous sensor sequences remains limited.

In response to the above limitations in temporal representation, deep learning-based time-series models have increasingly been applied to multi-sensor sequence modeling in fire scenarios in recent years. Li et al. [17] proposed a multi-sensor fusion indoor fire perception algorithm based on a Temporal Convolutional Network (TCN), which extracts dynamic features from sensor sequences through temporal convolution and performs classification by combining adaptive average pooling with a support vector machine. Liu et al. [18] proposed the EIF-LSTM model based on Long Short-Term Memory (LSTM), in which environmental variation information and environmental level information are fused before being fed into the LSTM to enhance the representation of fire-related temporal features; He et al. [19] further developed the BiLSTM-LN-SA model by integrating a bidirectional LSTM network, layer normalization, and self-attention, thereby strengthening the representation of key time-step features in multi-sensor fire detection. Jeong et al. [20] employed a Transformer encoder for indoor fire prediction based on sensor data, indicating that deep learning-based time-series models have gradually expanded from fire-state recognition to early warning tasks. However, these methods mainly perform modeling along the temporal or feature dimension and usually treat multi-node sensor inputs as ordinary multivariate time series, making insufficient use of the structural relationships among sensor nodes that arise from spatial layout, response order, and propagation paths.

For the intended application of cable-fire early warning in power cable tunnels, fire-related products such as particulate matter and gaseous species may propagate from the fire source to different monitoring positions, leading to differences in response timing, variation amplitude, and cross-node covariation among spatially distributed sensors. Therefore, discriminative information is not limited to instantaneous changes in a single indicator, but may also be reflected in spatial response patterns formed across multiple sensor nodes. Based on this consideration, the publicly available indoor EN 54 fire-test-room dataset reported in the source dataset paper [21] was selected for controlled experimental evaluation, because it contains cable-fire records collected by distributed multi-sensor nodes with known spatial deployment relationships. Although the experimental environment is different from an actual power cable tunnel, the dataset provides controlled multi-node cable-fire response records that are suitable for preliminary evaluation of the proposed spatial-response modeling strategy. Related studies using distributed multi-sensor nodes have further shown that sensor-node layout, distance from the fire source, and cross-node response differences affect the representation of early abnormal signals [21,22]. To characterize such node relationships, studies on graph neural networks and spatiotemporal graph modeling have shown that organizing sensor nodes or monitoring variables into graph structures can help explicitly represent dependencies among nodes and improve the model’s ability to capture complex spatiotemporal correlations [23,24,25,26]. Therefore, effectively exploiting spatial correlations among sensor nodes while extracting temporal evolution features remains a key challenge for multi-node cable-fire early prediction.

Based on the above analysis, cable fire early warning in power cable tunnel scenarios exhibits distinct engineering-specific characteristics. In relatively long, narrow, and enclosed cable routing spaces such as power cable tunnels, cables are typically arranged in a long-distance, dense, and continuous layout. Previous research on power cable tunnels indicates that cable-fire hazards are closely related to insulation aging, insulation breakdown, short-circuit faults, and the pyrolysis/combustion behavior of cable sheath and insulation materials [2]. During the incipient development of such fire events, abnormal sensor responses may not appear only as instantaneous abrupt changes. Fire-sensing studies and the source dataset paper indicate that fire-related indicators such as particulate matter, volatile organic compounds, and carbon monoxide can exhibit gradual temporal variations during early fire development, with gas-concentration changes occurring on the order of minutes rather than seconds in incipient low-heat-release scenarios [6,21]. In addition, the source dataset paper reported distance-dependent and sensor-node-dependent differences in the propagation behavior of PM, VOC, and CO, indicating that fire-related signals may vary across sensor locations in terms of response timing and amplitude [21]. Therefore, discrimination based solely on a single sensor or a single temporal dimension is insufficient to fully characterize the evolution of early-stage cable fire risk.

It should also be clarified that the point-type multi-sensor node approach investigated in this study is not intended to replace conventional linear cable fire detection systems used for long-distance continuous monitoring, such as linear heat detection or distributed temperature sensing systems. Instead, it is positioned as a complementary multi-parameter early-warning approach for key monitoring areas where distributed sensor nodes can be installed, such as cable joints, high-risk cable sections, equipment rooms, tunnel entrances, or local compartments. Compared with single-parameter threshold-based detection, the multi-sensor-node approach can capture early changes in particulate and gas-related indicators and provide additional data-driven evidence for identifying incipient cable-fire risks.

Considering these engineering-specific characteristics, this study constructs a cable fire early warning model based on multi-sensor spatiotemporal feature fusion. The model takes multi-indicator monitoring data collected by fixed sensor nodes within the 60 s preceding the current time t as input. Each sample is organized as a multi-node time series consisting of six consecutive time steps, nine sensor nodes, and five fire-related indicators, including PM0.5 concentration, PM1.0 concentration, volatile organic compound (VOC) concentration, total particulate matter, and carbon monoxide (CO) concentration. The cable fire state at t+180 s is taken as the target for early warning discrimination. In terms of model design, this study adopts ModernTCN as the temporal modeling backbone to extract the overall evolution trends and multi-scale variation features of multi-node monitoring signals within a short-term window. Considering that abnormal responses in the early stage of cable fires are usually weak, localized variations are inconspicuous, and such information may be attenuated during convolutional feature aggregation, a GRU module is introduced before the backbone to encode local fluctuations and short-term continuous changes between consecutive time steps. In addition, considering that spatially distributed sensor nodes in cable-tunnel-oriented monitoring applications have explicit deployment relationships, GCN+ is further introduced to model spatial correlations and cross-node coordinated responses among nodes. Accordingly, the proposed model incorporates indicator variations, temporal evolution, and spatial node responses during the development of cable fires into a unified early warning discrimination framework, thereby providing model support for cable fire risk identification in power cable tunnels.

The main contributions of this study are summarized as follows:

(1) An early cable fire prediction task is formulated based on multi-sensor time-series data. Instead of identifying only the current fire state, the proposed task uses the multi-sensor observations within the past 60 s to predict whether a cable fire will occur 180 s later, thereby extending cable fire recognition toward early warning of future fire risk.
(2) A multi-sensor spatiotemporal feature fusion model integrating GRU, ModernTCN, and GCN+ is proposed. In this framework, GRU is used to enhance local dynamic representation, ModernTCN is adopted to extract temporal evolution features from multivariate sensor sequences, and GCN+ is introduced to model spatial relationships among sensor nodes. The model therefore provides a joint representation of local dynamics, temporal evolution, and node relationships.
(3) The complementary effects of local dynamic enhancement and node relationship modeling are analyzed through comparative and ablation experiments. The results clarify how GRU and GCN+ enhance the ModernTCN backbone from the perspectives of short-term temporal dynamics and spatial relationship modeling, respectively.

## 2. Construction of a Cable Fire Early Warning Model Based on Multi-Sensor Spatiotemporal Feature Fusion

**Figure 1:**
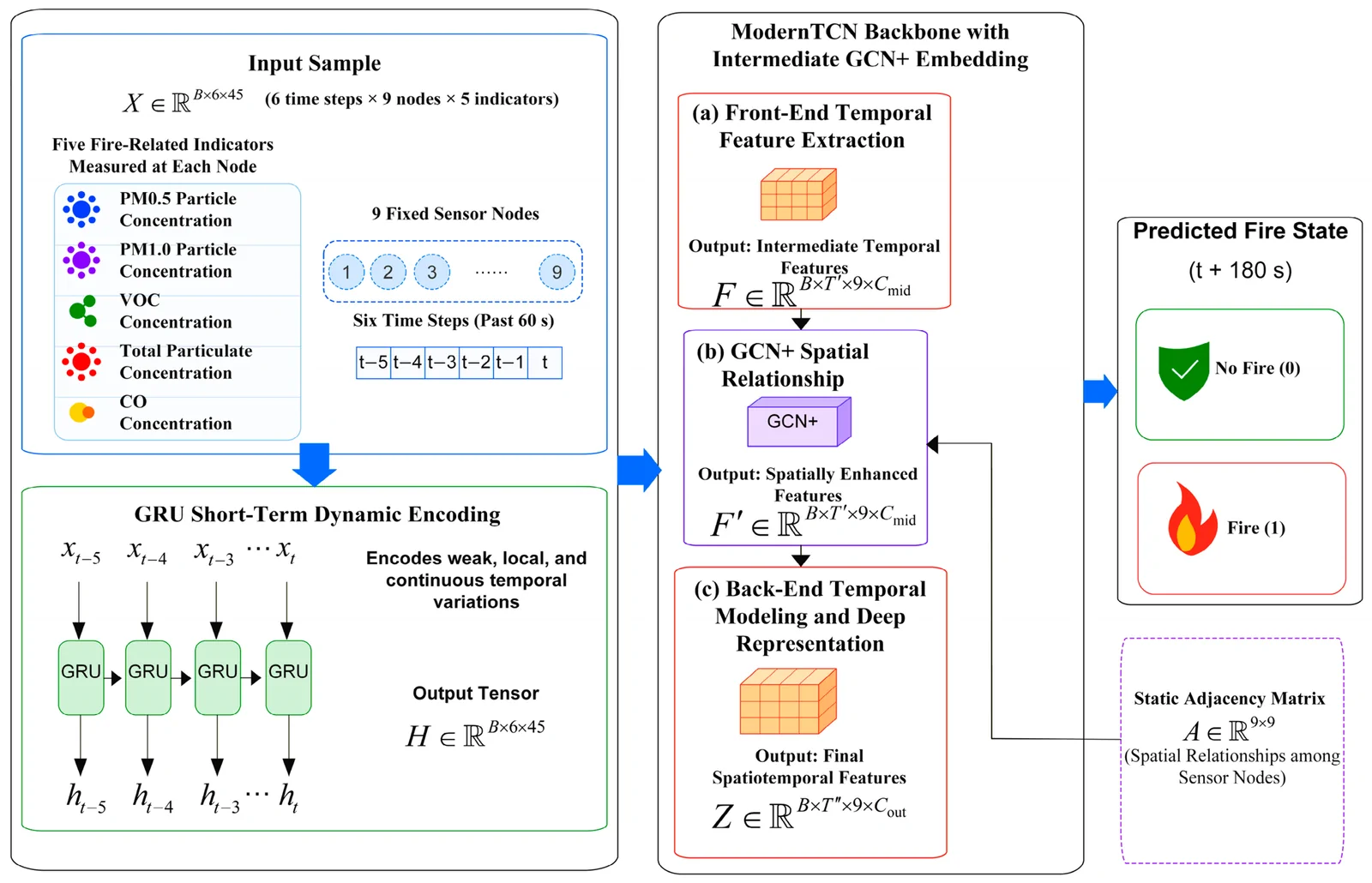
Overall architecture of the proposed model. Blue arrows indicate the main data and feature flow through the model, while the arrow from the static adjacency matrix to the GCN+ module denotes the input of spatial relationship information.

This study constructs a cable fire early warning model based on multi-sensor spatiotemporal feature fusion, and its overall structure is shown in Figure 1. The model takes multi-indicator monitoring data collected by fixed sensor nodes within the 60 s preceding the current time t as input. Each sample is represented as $X\in R^{B\times 6\times 45}$, where B denotes the batch size, 6 denotes six consecutive sampling steps with a 10 s sampling interval, and 45 corresponds to nine sensor nodes with five fire-related indicators at each node. The model predicts the fire/non-fire state at t+180 s.

In terms of structural design, the input sequence is first introduced into the GRU-based short-term dynamic encoding module. To enhance short-term local temporal variations while retaining the original multi-sensor observations, the GRU-encoded features are mapped back to the original feature space and fused with the input sequence through residual enhancement:

(1) $$H=LN\left(X+\alpha_{t}\Phi_{GRU}(X)\right)$$

where $\Phi_{GRU}(\cdot )$ denotes the GRU-based temporal encoding and feature mapping operation, LN(⋅) denotes layer normalization, and $\alpha_{t}$ is the temporal residual fusion coefficient. The fused representation H is then used as the input of the ModernTCN backbone. Through front-end temporal feature extraction, ModernTCN generates the intermediate temporal representation $F\in R^{B\times T^{\prime }\times 9\times C_{mid}}$, where $T^{\prime }$ denotes the temporal length of the intermediate feature representation and $C_{mid}$ denotes the number of intermediate feature channels.

To incorporate spatial dependencies among sensor nodes, GCN+ is embedded at the intermediate feature stage of the ModernTCN backbone. The intermediate representation F is reorganized into node-level features and introduced into the GCN+ module. Based on the static adjacency matrix $A\in R^{9\times 9}$, GCN+ performs spatial message passing among the nine sensor nodes and produces a spatial enhancement term $\Delta F_{GCN}$. The spatially enhanced representation is obtained through residual fusion:

(2) $$F^{\prime }=F+\alpha_{s}\Delta F_{GCN}$$

where $\alpha_{s}$ is the spatial residual fusion coefficient. This fusion strategy indicates that GCN+ does not replace the temporal representation extracted by ModernTCN; instead, it supplements the intermediate temporal features with spatial relationship information among sensor nodes. The enhanced representation $F^{\prime }\in R^{B\times T^{\prime }\times 9\times C_{mid}}$ is subsequently passed to the back-end temporal modeling component of ModernTCN to obtain the final spatiotemporal representation $Z\in R^{B\times T^{\prime \prime }\times 9\times C_{out}}$, where $T^{\prime \prime }$ and $C_{out}$ denote the temporal length and channel dimension of the final feature representation, respectively. Finally, Z is mapped to the binary fire/non-fire prediction result at t+180 s through the classification head.

### 2.1. GRU Short-Term Dynamic Encoding Module

**Figure 2:**
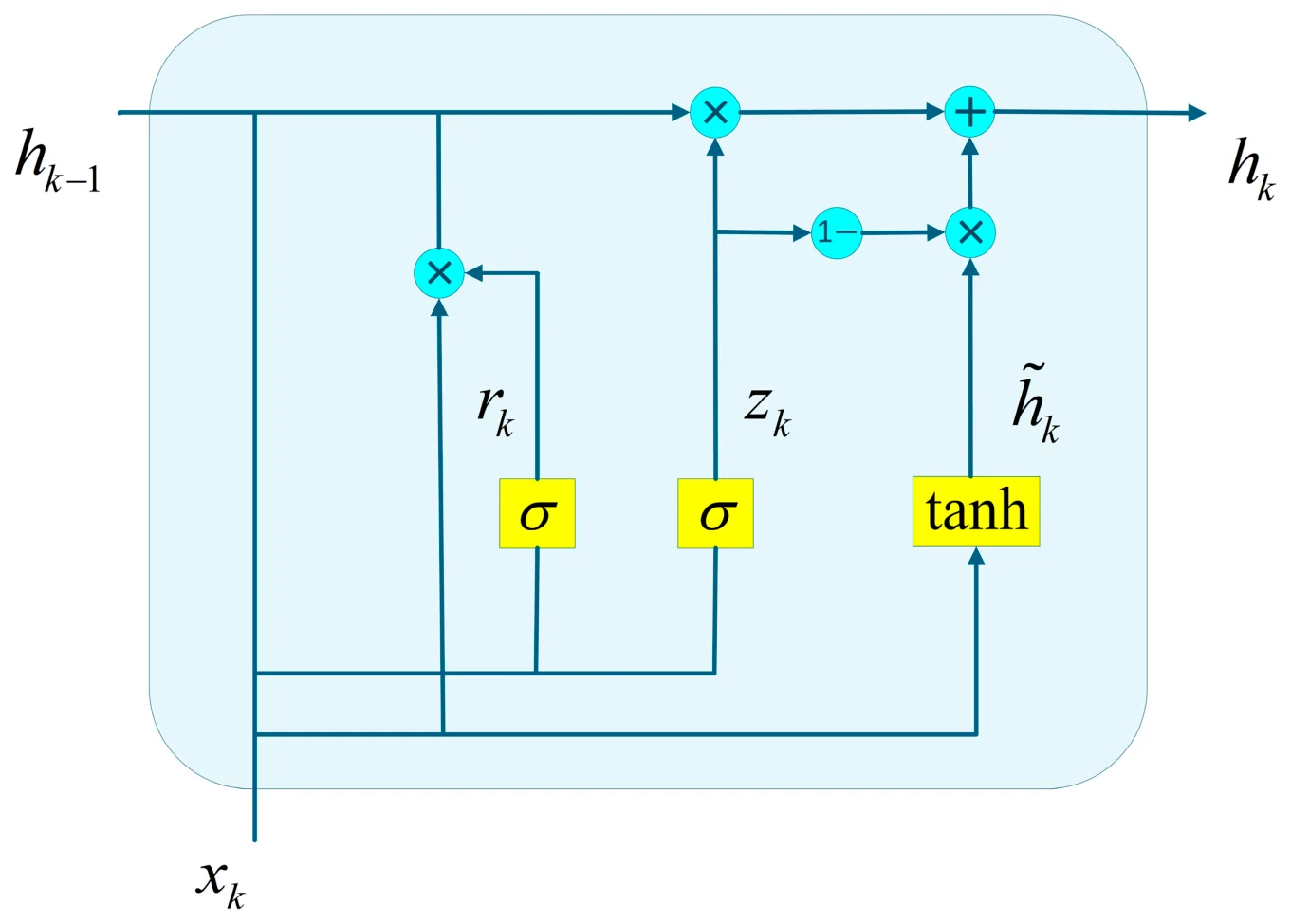
GRU structure.

The gated recurrent unit (GRU) was originally proposed by Cho et al. [27] and uses a gating mechanism to selectively retain historical state information in sequence modeling. In the cable fire early warning task for power cable tunnels, early-stage cable fires generally do not manifest as instantaneous abrupt changes. Instead, their precursor information is often reflected in the slow accumulation, local fluctuations, and short-term continuous changes in fire-related indicators, such as particulate matter, volatile organic compounds, and carbon monoxide, across consecutive sampling points. If the raw multi-sensor sequences are directly fed into the convolutional backbone, some local dynamic information with weak amplitudes or short durations may not be sufficiently represented. Therefore, this study introduces a GRU-based pre-encoding module in a serial manner before the ModernTCN temporal backbone to perform front-end encoding of short-term dynamic dependencies in the input sequence and to provide ModernTCN with a sequence representation containing local temporal context. The basic gating structure of the GRU is shown in Figure 2.

Let the model input be

(3) $$X_{in}\in R^{B\times L\times M}$$

where B denotes the batch size, L denotes the input sequence length, and M denotes the multi-sensor feature dimension at each time step. In this task, L=6 corresponds to 6 sampling points over the past 60 s, and M=45 corresponds to 5 monitoring indicators for each of the 9 sensor nodes. For a single sample, the input sequence can be represented as

(4) $$X=\{x_{1},x_{2},\ldots ,x_{L}\},\;x_{k}\;\in R^{M},\;k=1,2,\ldots ,L$$

where $x_{k}$ denotes the multi-sensor observation vector at the k-th time step. For the k-th time step, the calculation process of GRU is as follows.

(1) Reset Gate Calculation

The reset gate controls how much historical information in the hidden state from the previous time step is involved in calculating the current candidate hidden state, and is expressed as

(5) $$r_{k}=\sigma (W_{r}x_{k}+U_{r}h_{k-1\;}+b_{r})$$

where $r_{k}$ denotes the reset gate at the k-th time step, $h_{k-1}$ denotes the hidden state at the previous time step, $W_{r}$ and $U_{r}$ are weight matrices, $b_{r}$ is the bias term, and σ(⋅) denotes the sigmoid activation function. For early warning of cable fires, the reset gate regulates the influence of the existing state information from the previous sampling time on the current candidate state, allowing the model to process short-term fluctuations without relying entirely on historical states.

(2) Update Gate Calculation

The update gate controls the balance between retaining the previous hidden state and incorporating the current candidate hidden state, and its expression is given as

(6) $$z_{k}=\sigma (W_{z}x_{k}+U_{z}h_{k-1}+b_{z})$$

where $z_{k}$ denotes the update gate at the k-th time step, $W_{z}$ and $U_{z}$ are weight matrices, and $b_{z}$ is the bias term. Because sensor responses in the early stage of cable fires usually exhibit gradual accumulation, the update gate can retain useful historical state information across consecutive time steps while incorporating new local variations from the current sampling point.

(3) Candidate Hidden State Calculation

The candidate hidden state is used to represent the new information formed by the current input and the historical state filtered by the reset gate, and its expression is given as

(7) $${\tilde{h}}_{k}=\tanh(W_{h}x_{k}+U_{h}(r_{k}⊙h_{k-1})+b_{h})$$

where ${\tilde{h}}_{k}$ denotes the candidate hidden state, $W_{h}$ and $U_{h}$ are weight matrices, $b_{h}$ is the bias term, tanh(⋅) denotes the hyperbolic tangent activation function, and ⊙ denotes element-wise multiplication.

(4) Hidden State Update

The hidden state at the current time step is obtained by weighting the hidden state from the previous time step and the current candidate hidden state:

(8) $$h_{k}=z_{k}⊙h_{k-1}+(1-z_{k})⊙{\tilde{h}}_{k}$$

where $h_{k}$ denotes the output hidden state at the k-th time step. Through the above gating calculations, GRU can dynamically determine the information to retain, forget, and update based on the current input and historical state, thereby capturing short-term dynamic changes in the sensor sequence.

After GRU encoding, the hidden representation sequence $H^{GRU}$, which contains temporal context information, can be obtained as

(9) $$H^{GRU}=\{h_{1},h_{2},\ldots ,h_{L}\}$$

This representation encoded by GRU provides local temporal context for the subsequent ModernTCN backbone, allowing the model to first capture fine-grained temporal dependencies at the front end, while ModernTCN further extracts deeper multi-scale temporal features from the encoded sequence.

### 2.2. ModernTCN Temporal Backbone

To extract the main temporal features in the cable fire early warning task for power cable tunnels, this study adopts ModernTCN as the temporal modeling backbone. ModernTCN is a pure convolutional architecture for multivariate time-series analysis. Compared with traditional TCN architectures, which are limited by a relatively small receptive field, ModernTCN expands the effective receptive field through large-kernel convolution and structural reparameterization, thereby enhancing its ability to represent variation patterns in multivariate time series while maintaining high computational efficiency [28]. For the cable fire early warning task investigated in this study, discriminative information is usually not derived only from sensor readings at a single time step, but is more prominently reflected in the continuous variations, overall evolution trends, and multi-scale variation features of multi-node and multi-indicator monitoring signals within the past 60 s observation window. Therefore, ModernTCN is suitable as the temporal backbone of the proposed model for extracting temporal evolution features related to the development of cable fires from the front-end encoded multi-sensor sequence.

**Figure 3:**
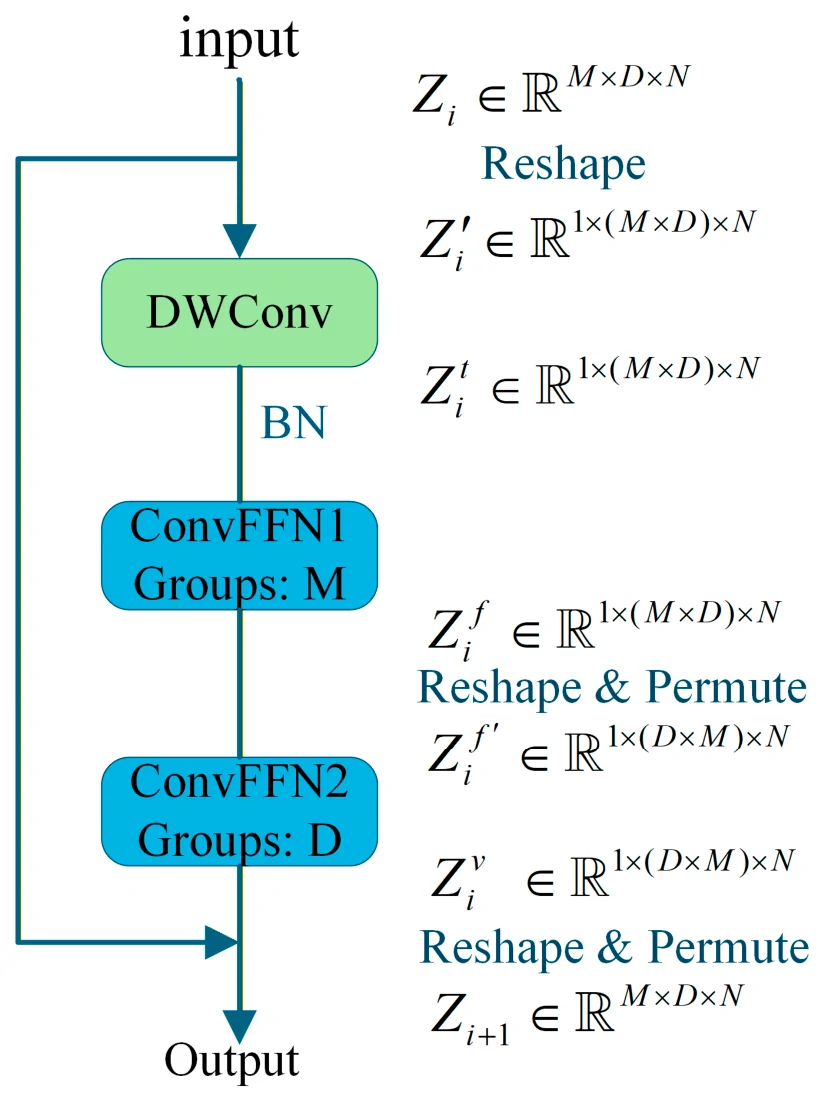
Architecture of the ModernTCN temporal modeling backbone.

The core design of ModernTCN is built on a decoupled architecture, which handles temporal dependency modeling, feature mixing, and variable interaction separately, as shown in Figure 3. Each ModernTCN block mainly consists of three key components: a depthwise convolution layer (DWConv), grouped pointwise convolution feed-forward module 1 (ConvFFN1), and grouped pointwise convolution feed-forward module 2 (ConvFFN2). Among these components, DWConv performs independent convolutions along the temporal dimension on the feature channels of each variable to model temporal dependencies; ConvFFN1 performs intra-variable feature mixing; and ConvFFN2 enables information interaction among different variables. Such modular decomposition reduces computational overhead while preserving model expressiveness. In particular, DWConv adopts large-kernel convolution, which enhances temporal modeling capability by expanding the effective receptive field, without relying on excessive layer stacking to capture longer temporal context. For the cable fire early warning task considered in this study, DWConv helps capture the continuous evolution process of multi-sensor signals within the past 60 s short-term observation window, while ConvFFN1 and ConvFFN2 further perform intra-variable feature mixing and inter-variable information interaction. This enables the model to extract overall evolution trends and multi-scale variation features from the temporal changes in multiple fire-related indicators.

The ModernTCN backbone propagates features layer by layer through residual connections, and its forward propagation can be expressed as follows:

(10) $$Z_{i+1}=\;Block(Z_{i})+Z_{i}$$

where $Z_{i}\in R^{M\times D\times N}$ denotes the input tensor of the i-th block, and M, D, and N denote the number of variables, feature dimension, and number of time steps, respectively. Block(⋅) denotes the nonlinear mapping process jointly composed of DWConv, normalization, ConvFFN1, ConvFFN2, and tensor rearrangement. This residual structure enables each block to extract higher-level temporal features while preserving effective underlying information from the input, thereby improving the stability of network training.

### 2.3. Embedded GCN+ Spatial Relationship Enhancement Module

In cable fire early warning scenarios in power cable tunnels, sensor nodes are typically deployed at fixed positions along the tunnel direction. When smoke generated during the early stage of a cable fire diffuses within the tunnel, its propagation is affected by the propagation path and the spatial positions of sensor nodes, resulting in differences among nodes in response timing, variation amplitude, and coordinated fluctuations. Therefore, feature extraction based solely on the temporal dimension makes it difficult to explicitly exploit the spatial correlations embedded in multi-node monitoring signals. Based on this consideration, this study introduces the GCN+ module proposed by Luo et al. [29] and embeds it at the intermediate feature stage of ModernTCN to construct a spatial relationship enhancement module. This module supplements the model’s ability to capture spatial relationships among sensor nodes, thereby representing spatial correlations and cross-node coordinated responses among fixed sensor nodes.

**Figure 4:**
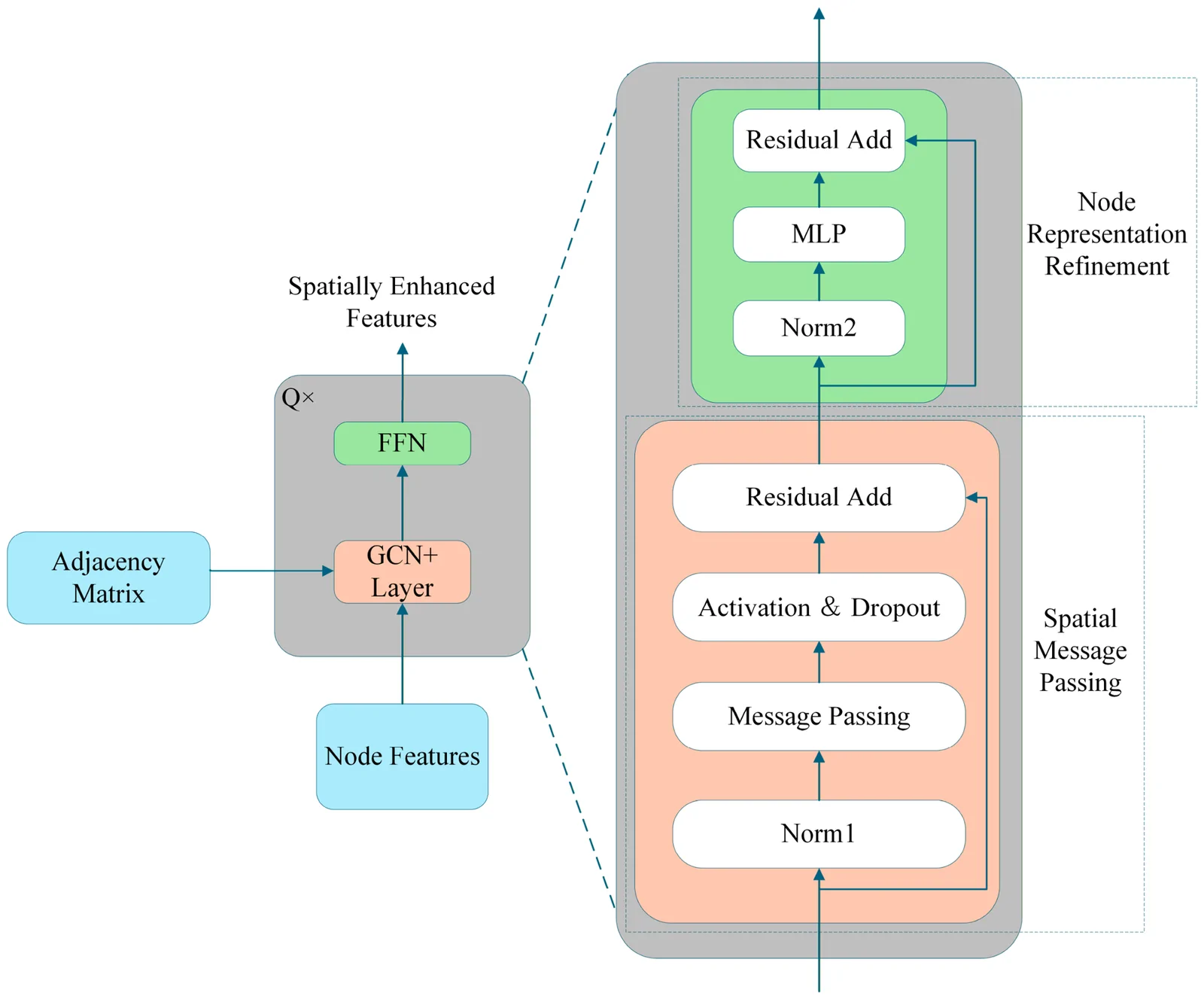
Structure of the embedded GCN+ spatial relationship enhancement module.

As shown in Figure 4, the embedded GCN+ spatial relationship enhancement module mainly comprises three parts: a static adjacency matrix, node feature construction, and stacked graph encoding blocks. Each graph encoding block consists of a GCN+ layer and a feed-forward network (FFN). The former is responsible for message propagation and node relationship modeling based on the graph structure, whereas the latter further applies nonlinear transformation to the propagated node representations. The internal structure of a single graph encoding block is shown on the right side of Figure 4.

To explicitly model node spatial relationships in the intermediate feature representation produced by ModernTCN, this study treats each sensor node as a graph node and reorganizes the multivariate intermediate representation into node-level representations. After reorganization, the intermediate feature representation corresponding to the i-th sensor node is denoted as

(11) $$\begin{array}{c} Z^{\left(i\right)}\in R^{K\times D\times P},\;\;i=1,2,\ldots ,V \end{array}$$

where V denotes the number of sensor nodes, K denotes the number of monitoring indicators contained in each node, D denotes the intermediate feature dimension, and P denotes the temporal dimension after patching.

Considering that this module is mainly used to supplement the spatial dependencies among sensor nodes, this study performs average aggregation on the reorganized intermediate features along the patched temporal dimension to obtain node-level representations:

(12) $$s^{\left(i\right)}={\;Mean}_{P}\left(Z^{\left(i\right)}\right)$$

where ${Mean}_{P}(\cdot )$ denotes average aggregation along the patched temporal dimension. This node representation characterizes the overall response state of the i-th sensor node within the current window and is then passed through linear mapping to serve as the node input to the graph encoding block.

In the graph structure setting, this study uses a static adjacency matrix A to describe the spatial proximity relationships among sensor nodes. The adjacency matrix was constructed based on the spatial coordinates of the nine sensor nodes in the EN 54 test room reported in the source dataset paper [21]. Following the distance definition used in the source dataset paper, Manhattan distance was adopted to characterize the spatial relationship between sensor nodes. Let $p_{i}=(x_{i},y_{i},h_{i})$ and $p_{j}=(x_{j},y_{j},h_{j})$ denote the spatial coordinates of sensor nodes i and j, respectively. The Manhattan distance between two nodes was calculated as

(13) $$d_{ij}=|x_{i}-x_{j}|+|y_{i}-y_{j}|+|h_{i}-h_{j}|$$

The use of Manhattan distance follows the rationale of the source dataset paper [21]. Specifically, the source study selected Manhattan distance rather than Euclidean distance to more precisely evaluate the plume-shaped propagation behavior of released combustion products in the EN 54 test room. Euclidean distance may yield similar values for sensor nodes with different horizontal offsets from the source, whereas Manhattan distance better captures the varying x–y displacement components between the source and the sensor nodes. Therefore, in this study, the Manhattan-distance-based adjacency matrix was used to encode the spatial proximity relationship among the sensor nodes in the source experimental room.

To preserve node-specific information and stabilize the message-passing process, self-connections were included in the adjacency matrix, and the resulting matrix was row-normalized to obtain the graph propagation matrix $\tilde{A}$. This operation enables each node to retain its own features while aggregating information from adjacent nodes and reduces the influence of differences in node connectivity on the propagation results.

On this basis, GCN+ performs node relationship modeling on node representations by stacking graph encoding blocks. Let the input of the q-th graph encoding block be $H^{\left(q\right)}$, where q = 0,1,…,Q−1, and Q denotes the number of stacked graph encoding blocks. The update process can then be written as follows:

(14) $$\begin{array}{ll} {\hat{H}}^{\left(q\right)} & ={GCN}^{+}\left(H^{\left(q\right)},\tilde{A}\right)\\ H^{\left(q+1\right)} & ={\hat{H}}^{\left(q\right)}+FFN\left(Norm\left({\hat{H}}^{\left(q\right)}\right)\right) \end{array}$$

where ${GCN}^{+}(\cdot )$ denotes the message propagation and node relationship modeling process based on the graph propagation matrix, ${\hat{H}}^{\left(q\right)}$ denotes the node representation updated by the GCN+ layer, Norm(⋅) denotes the normalization operation, and FFN(⋅) denotes the feed-forward network. Through the above update, the node representations can retain their own information while integrating spatial relationship information transmitted from adjacent nodes, thereby obtaining more discriminative node relationship representations.

The node relationship representations obtained through graph encoding are further integrated into the intermediate feature representations of ModernTCN, enabling the model to incorporate spatial relationship information among sensor nodes while maintaining the temporal modeling capability of the temporal convolutional backbone. Accordingly, the embedded GCN+ spatial relationship enhancement module enhances the model’s ability to represent cross-node coordinated variations during the early stage of fire and provides more informative spatiotemporal feature representations for subsequent classification prediction.

## 3. Experimental Settings and Result Analysis

### 3.1. Dataset Source and Preprocessing

The experimental data used in this study were obtained from the publicly available Indoor Fire Dataset with Distributed Multi-Sensor Nodes [30]. The experimental setup and data acquisition procedure of this dataset are reported in detail in the accompanying dataset paper [21]. The dataset was collected in an indoor EN 54 fire test room and contains spatially distributed multi-sensor measurements under fire conditions, non-fire nuisance conditions, and normal background conditions. Compared with datasets collected using a single sensor or a single fire scenario, this dataset provides multi-node deployment, multiple types of sensor responses, and continuous multivariate time-series records, making it suitable for evaluating early fire detection models based on spatiotemporal sensor information.

The hardware configuration of the sensor nodes is introduced here to clarify the physical basis of the multi-sensor measurements. According to the source dataset paper [21], each sensor node was controlled by an ESP32 microcontroller, and the sensor readings were transmitted to a Raspberry Pi server through WiFi using the MQTT protocol. The received data were decoded and stored in an Influx time-series database with UTC timestamps, enabling synchronized multi-node time-series recording under different experimental conditions. Each node integrated particulate, gas, optical, and environmental sensors, as summarized in Table 1.

The same source further reports that the CO/MF-1000 sensor has a T90 response time of approximately 25 s, while the UST6xxx sensor array operates with internal temperature cycles of approximately 10 s for H_2_ detection. The dataset records sensor measurements at a sampling interval of 10 s.

**Figure 5:**
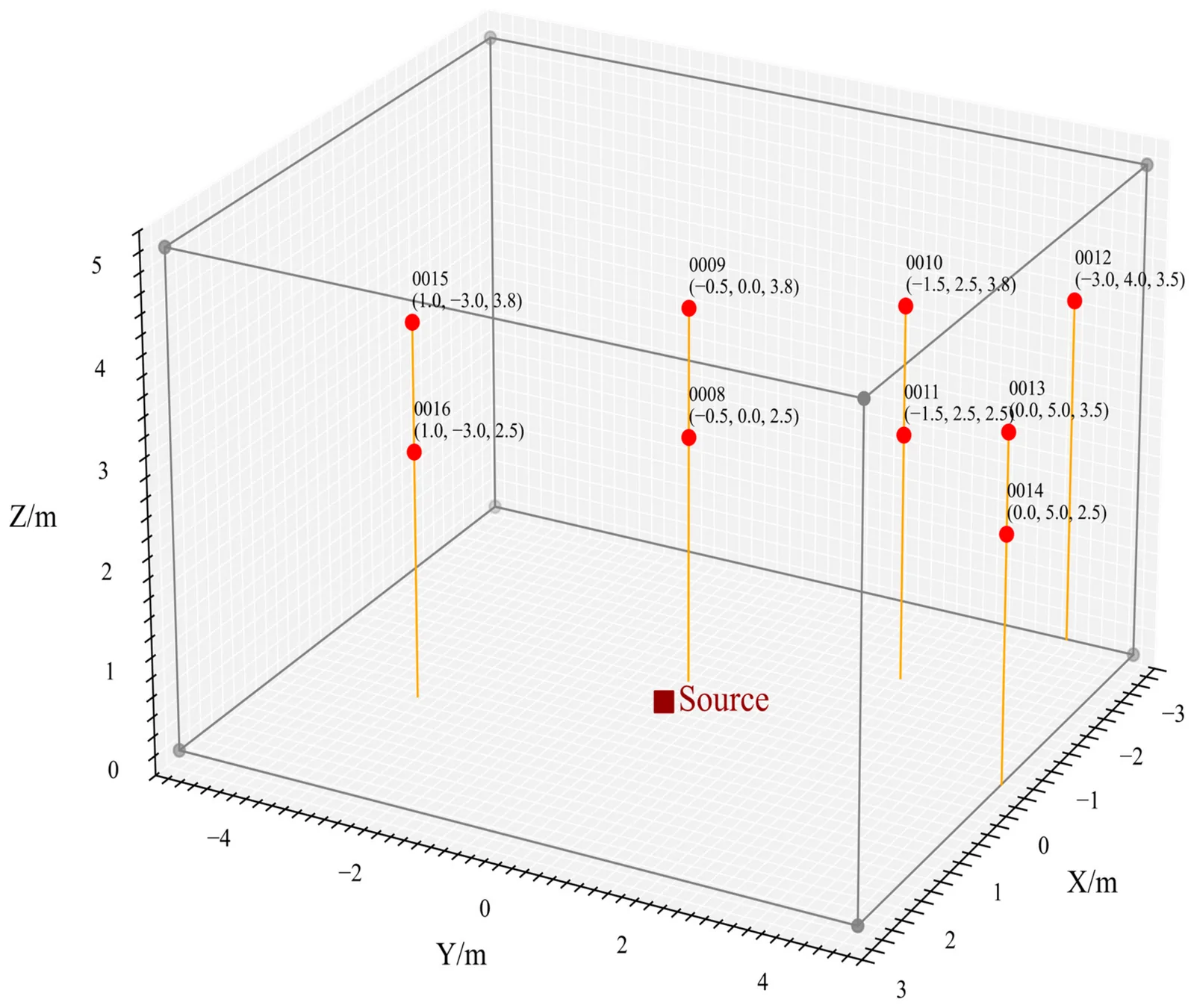
Spatial arrangement of the fire source and the nine sensor nodes in the EN 54 indoor test room. Reprinted from Ref. [21] under the Creative Commons Attribution 4.0 International (CC BY 4.0) license.

In the source dataset, the experiments were conducted in a non-ventilated EN 54 fire test room with dimensions of 7 m × 10 m × 4 m. The sensor network consisted of nine sensor nodes, numbered from 0008 to 0016, which were placed at different horizontal positions and heights around the fire source. The exact spatial coordinates and the corresponding Euclidean and Manhattan distances from the fire source were reported in the source dataset paper [21]. This spatial arrangement enabled the dataset to record differences in sensor responses caused by the distance between the emission source and each sensor node, and it also provided the spatial basis for constructing the distance-based adjacency matrix used in the GCN+ module. The spatial arrangement of the fire source and sensor nodes is shown in Figure 5.

The public dataset is structured as a continuous multivariate time series. The source dataset paper reports that the dataset contains 305,304 rows and 16 columns, where each row corresponds to the measurements of one sensor node at a specific timestamp. The original experiments include four fire scenarios, namely wood fire, candle fire, cable fire, and cotton-lint fire, and three non-fire nuisance scenarios, namely ethanol, deodorant, and hairspray disturbances. The fire experiments were repeated three times for each fire type, while the nuisance experiments included three ethanol tests, two deodorant tests, and one hairspray test. Background measurements were recorded between fire and nuisance experiments after room ventilation and a waiting period.

The dataset provides several types of labels, including a scenario-specific label, a binary anomaly label distinguishing background from anomaly events, a ternary label distinguishing background, nuisance, and fire events, and a progress label describing event progression. Among the 305,304 records in the original dataset, 253,327 are labeled as background (82.9753% of the total) and 51,977 as anomalies (17.0247% of the total). The anomaly records comprise 38,148 fire-anomaly records (12.4951% of the total) and 13,829 nuisance-anomaly records (4.52958% of the total). These labels make the dataset suitable for different fire-detection tasks, including binary fire detection, nuisance rejection, and scenario-specific analysis.

Since this study focuses on cable fire early warning for power cable tunnel applications, the cable-fire-related records were selected from the public dataset for subsequent sample construction. In the source dataset, the cable fire scenario was repeated three times. The source dataset paper reports that the cable fire was performed using a halogen-free NHXMH-type cable with a length of 35 cm, and the fire was triggered by an electrical overload of approximately 130 A DC. The cable was insulated on the lower side with rock wool, and the experiments were conducted in the non-ventilated EN 54 test room. In the processed cable-fire data used in this study, the three cable fire records lasted approximately 48 min, 51 min, and 47 min, corresponding to 290, 309, and 284 unique timestamps, respectively, at the 10 s sampling interval.

In the source dataset paper, the start of each experiment was used as $t_{0}$ for analyzing the time delay between the experimental onset and the threshold exceedance of different sensor measurements. For the cable-fire scenario, $t_{0}$ corresponds to the onset of the cable-fire experiment, in which the cable was subjected to electrical overload, rather than the threshold exceedance of any individual sensor. Therefore, in this study, the start of a cable-fire record is interpreted as the experimental onset/electrical-overload initiation. For the binary prediction task, fire-anomaly data points were determined according to the fire label provided in the source dataset: data before $t_{0}$ correspond to the background/non-fire condition, whereas data from $t_{0}$ onward are labeled as fire-anomaly data points. The physical subprocess labels, such as outgassing, smoldering, glowing, end of experiment, and ventilating, are used only to describe the evolution of the cable-fire process and are not treated as separate prediction classes.

**Figure 6:**
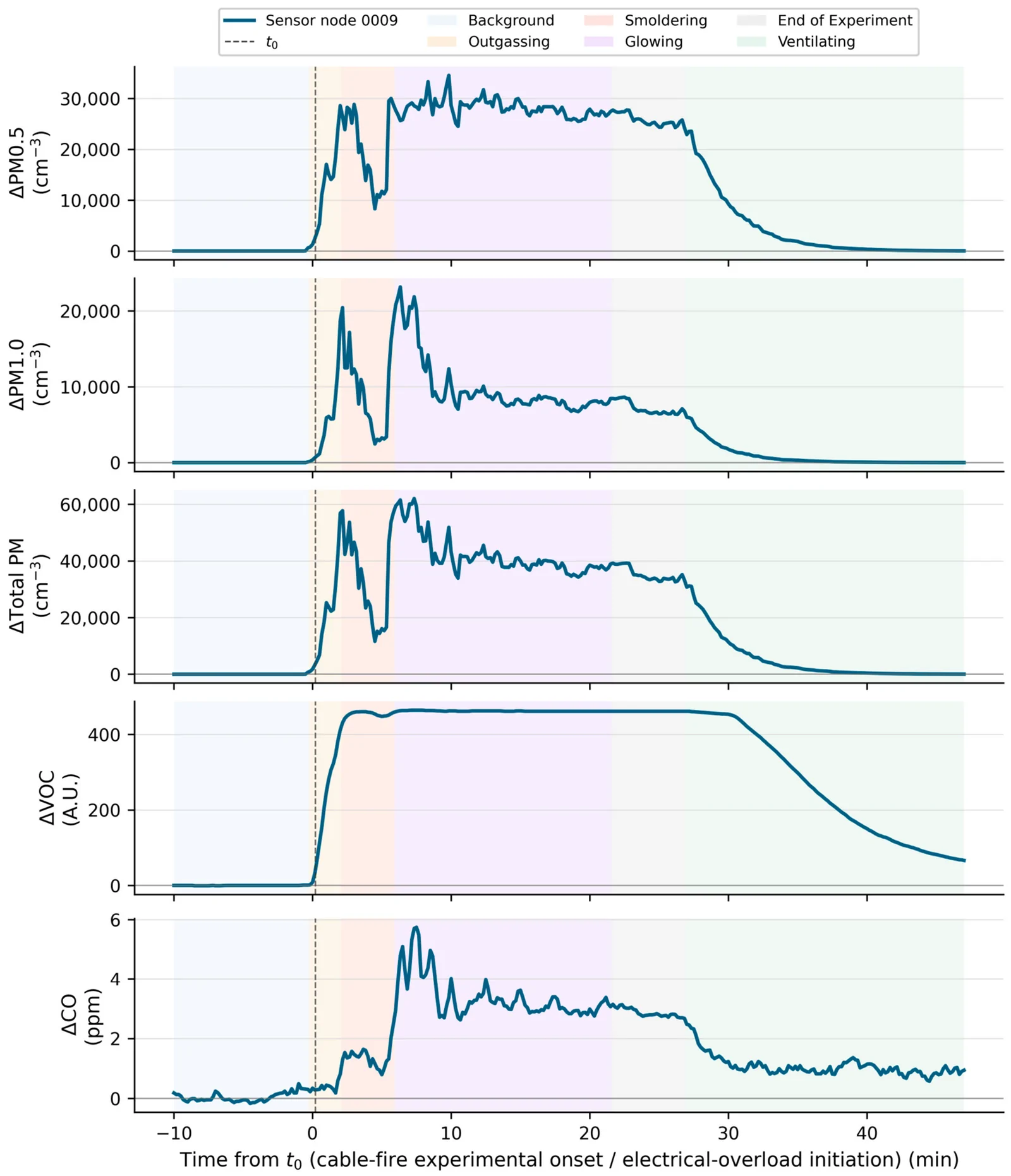
Example temporal evolution of selected indicators in cable-fire experiment E3 at sensor node 0009.

As shown in Figure 6, the selected indicators do not exhibit a single instantaneous step change after $t_{0}$ but show stage-dependent temporal response patterns during the cable-fire process. According to the source dataset paper, smoldering corresponds to a flameless decomposition/combustion-related stage, whereas glowing is associated with a later carbonization/incandescent process induced by electrical-overload heating of the cable insulation. These temporal variations provide direct time-series evidence for the use of multi-indicator observations in early cable-fire warning.

Considering the response characteristics of particulate matter and gaseous products during the early stage of cable fires, this study selected PM0.5, PM1.0, volatile organic compound (VOC), total particulate matter, and carbon monoxide (CO) as the model input indicators. These indicators represent early fire-related responses from the perspectives of fine particulate release, volatile gas variation, and combustion product accumulation. At each sampling time, the five selected indicators were collected from nine fixed sensor nodes; therefore, a single time step was represented by a 45-dimensional multi-node and multi-indicator observation vector. Based on the 10 s sampling interval, cable-fire early warning samples were further constructed using a sliding window of six consecutive sampling steps, corresponding to the multi-sensor observations within the 60 s preceding the current time t. Each sample was represented as a sequence of six 45-dimensional observations, and its label was assigned according to the binary fire/non-fire state at the single future time point t + 180 s. Thus, the prediction target corresponds to a fixed-horizon future state rather than to the occurrence of fire at any time within a 180 s future interval. After sample construction, the dataset was divided into training, validation, and test sets. The resulting training, validation, and test sets contained 1199, 257, and 258 samples, respectively. Specifically, the training set included 581 non-fire samples and 618 fire samples, the validation set included 125 non-fire samples and 132 fire samples, and the test set included 125 non-fire samples and 133 fire samples.

### 3.2. Experimental Parameter Settings and Evaluation Metrics

To evaluate the effectiveness of the proposed model in the cable fire early warning task, comparative experiments and ablation studies were conducted. In the comparative experiments, LSTM, GRU, and TCN were selected as typical temporal modeling baselines to evaluate the performance of the proposed model against commonly used recurrent neural networks and convolutional temporal models. A module-level ablation study was conducted to evaluate the individual and joint contributions of the GRU short-term dynamic encoding module and the GCN+ spatial relationship enhancement module. ModernTCN was used as the temporal backbone baseline, and GRU, GCN+, and their combination were introduced on this basis, forming four experimental settings: ModernTCN, ModernTCN + GRU, ModernTCN + GCN+, and the complete model (Ours). These settings were used to analyze the influence of different modules on model performance. In all experiments, each model took sequences of five fire-related indicators collected by nine fixed sensor nodes within the 60 s preceding the current time t as input, with an input dimension of 6 × 45 for each sample. The model output was the binary classification result of the cable-fire state at the single future time point t + 180 s, namely the non-fire state or the fire state. Each constructed sample was evaluated independently at this prediction horizon; the evaluation was not performed at the level of an entire experimental record. The 180 s prediction horizon was selected because fire-sensing studies and the source dataset paper indicate that early fire-related particulate and gas responses, such as PM, VOC, and CO, may evolve on a minute scale rather than as second-level instantaneous changes during incipient low-heat-release fire development [6,21]. Therefore, this setting provides a minute-level early-warning interval while maintaining a direct temporal association between the preceding 60 s multi-sensor observations and the subsequent cable-fire state.

Model training was performed using the Adam optimizer with an initial learning rate of 0.0001, and cross-entropy loss was adopted as the loss function. The maximum number of training epochs was set to 80, the batch size was set to 64, and an early stopping strategy based on the validation-set F1-score was applied, with the patience value set to 12. To reduce the influence of randomness on the experimental results, the random seed was fixed at 2021. All models were trained and evaluated using the same training, validation, and test set splits to ensure fair and consistent comparisons among different models.

For performance evaluation, this study adopted Accuracy, true positive rate (TPR), false negative rate (FNR), false positive rate (FPR), F1-score, and area under the receiver operating characteristic curve (AUC) to comprehensively evaluate the binary classification performance of different models. In this binary classification task, the fire state was defined as the positive class, and the non-fire state was defined as the negative class. Accordingly, true positive (TP) denotes fire samples correctly predicted as fire, true negative (TN) denotes non-fire samples correctly predicted as non-fire, false positive (FP) denotes non-fire samples incorrectly predicted as fire, and false negative (FN) denotes fire samples incorrectly predicted as non-fire. For the fixed-threshold metrics, including Accuracy, TPR, FNR, FPR, Precision, and F1-score, the predicted class label was determined by selecting the class with the highest softmax probability.

The evaluation metrics were calculated as follows:

(15) $$Accuracy=\frac{TP+TN}{TP+TN+FP+FN}$$

(16) $$TPR=\frac{TP}{TP+FN}$$

(17) $$FNR=\frac{FN}{TP+FN}$$

(18) $$FPR=\frac{FP}{FP+TN}$$

(19) $$Precision=\frac{TP}{TP+FP}$$

(20) $$F1-score=\frac{2\times Precision\times TPR}{Precision+TPR}$$

(21) $$AUC=\int_{0}^{1}TPR(FPR)\;dFPR$$

Accuracy reflects the overall proportion of correctly classified samples. TPR, also known as recall or sensitivity, represents the proportion of fire samples correctly identified by the model, whereas FNR represents the missed-detection rate for fire samples. FPR represents the false-alarm rate for non-fire samples. Precision is used to calculate F1-score and represents the proportion of predicted fire samples that are truly fire samples. F1-score evaluates the balance between Precision and TPR under the fixed classification results. AUC measures the overall discriminative ability of the model based on the receiver operating characteristic curve, which describes the relationship between TPR and FPR under different decision thresholds.

### 3.3. Results and Analysis

To evaluate the effectiveness of the proposed model in the cable-fire early-warning task under the selected laboratory dataset setting, this study compares it with three typical temporal modeling methods: LSTM, GRU, and TCN. Considering that Accuracy, TPR, FNR, FPR, F1-score, and AUC reflect overall classification correctness, fire-sample detection capability, missed-detection risk, false-alarm risk, the balance between precision and recall, and discriminative ability under different decision thresholds, respectively, the comparative results are presented in Table 2.

As shown in Table 2, compared with the three typical temporal models, namely LSTM, GRU, and TCN, the proposed method achieves better overall performance on the test set. In terms of Accuracy, the proposed method reaches 0.9884, which is higher than that of LSTM, the best-performing baseline model, with an Accuracy of 0.9612, corresponding to an improvement of 2.72 percentage points. The proposed method also achieves the highest F1-score of 0.9887 and the highest AUC of 0.9994, indicating advantages in both the balance between precision and recall and the discriminative ability under different decision thresholds.

For the early cable fire prediction task, reducing the risk of missed detections is of greater practical engineering significance than merely improving overall Accuracy. As shown in Table 2, among LSTM, GRU, and TCN, TCN yields the lowest FNR, with a value of 0.0602; in contrast, the FNR of the proposed method is further reduced to 0.0150. Meanwhile, the proposed method maintains a low FPR of 0.0080, indicating that the reduction in missed detections is not accompanied by a substantial increase in false-alarm risk. The TPR of the proposed method reaches 0.9850, indicating that the model can identify fire samples more effectively and reduce the risk of missed detections.

The results indicate that LSTM, GRU, and TCN can model temporal dependencies in sensor sequences, but they still present a certain risk of missed detections in this multi-node fire prediction task. The performance advantage of the proposed method may be associated with the further introduction of local dynamic enhancement and node relationship modeling on top of the temporal backbone, which enables the model to use early-stage local variations and cross-node coordinated response information. The specific contributions of the related modules are further analyzed in the subsequent ablation experiments.

The incorrectly predicted samples of the proposed model were further examined based on the test-set classification results. The errors accounted for only a small proportion of the test set, indicating that most fire and non-fire samples were correctly discriminated. The remaining errors mainly corresponded to boundary-like samples with overlapping sensor-response patterns between fire and non-fire conditions, where local fluctuations in particulate or gas-related indicators may resemble early fire-related variations. Such samples can reduce the separability between the two classes at individual decision points. Nevertheless, the proposed model maintained both a low missed-detection rate and a low false-alarm rate on the test set, indicating that these few errors did not reflect a systematic tendency toward either missed fire detection or excessive false alarms.

In addition, considering the inference-efficiency requirements of practical early warning systems, the single-sample inference time of the proposed model during the test phase was further measured. Under the current experimental settings, the average inference time of the proposed model is 0.164585 ms per sample, indicating that it achieves good inference efficiency while maintaining high prediction performance.

**Figure 7:**
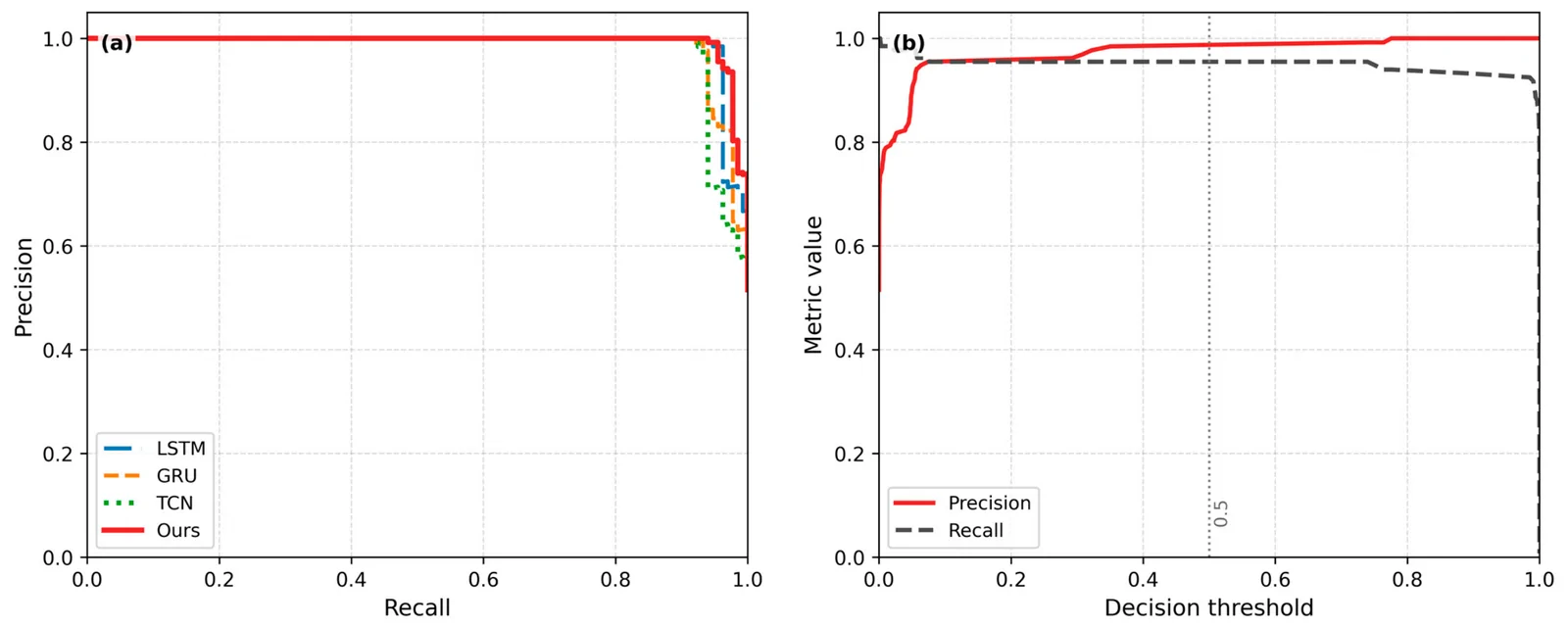
PR-curve and threshold-dependent performance comparison. (**a**) PR curves of different models with different line styles. (**b**) Precision and recall of the proposed model under different decision thresholds.

To further analyze the recognition performance of different models for fire samples under different decision thresholds, the Precision–Recall (PR) curves of LSTM, GRU, TCN, and the proposed model are plotted, as shown in Figure 7a. In the PR-curve analysis, each model outputs a predicted probability for the fire class, and the decision threshold applied to this probability is varied from 0 to 1. For each threshold, the corresponding precision and recall are calculated, and the PR curve is obtained by plotting precision against recall across all thresholds. Accordingly, the PR curve provides a direct visualization of the trade-off between precision and recall for fire-sample recognition under different threshold settings. To further illustrate the threshold-dependent behavior of the proposed model, Figure 7b shows the changes in precision and recall when the decision threshold varies from 0 to 1.

As shown in Figure 7a, in the high-recall region, the PR curve of the proposed model generally lies above those of LSTM, GRU, and TCN. This indicates that the proposed model can maintain relatively high precision while preserving a high fire-sample detection rate. For the early cable fire prediction task, recall reflects the detection capability for fire samples, whereas precision reflects the reliability of fire-alarm predictions. In addition, Figure 7b shows that the proposed model maintains a stable precision–recall trade-off over a broad range of decision thresholds. These results indicate that the proposed model has more robust positive-class recognition performance under different threshold settings.

To further evaluate the contributions of the GCN+ and GRU modules to the performance improvement of the model, the ablation study adopted ModernTCN as the backbone baseline. On this basis, the GCN+ module, the GRU module, and their combination were respectively incorporated to form four models, namely ModernTCN, ModernTCN + GCN+, ModernTCN + GRU, and the complete model (Ours), thereby verifying the contribution of each module. Specifically, the comparison between ModernTCN and ModernTCN + GRU evaluates the effect of the GRU module, the comparison between ModernTCN and ModernTCN + GCN+ evaluates the effect of the GCN+ module, and the comparison with Ours evaluates the joint effect of the two modules. The experimental results are presented in Table 3.

The ablation study results in Table 3 show that, compared with the ModernTCN backbone baseline model, the independent incorporation of either the GCN+ module or the GRU module improves the classification performance to a certain extent. When the two modules are incorporated simultaneously, the complete proposed model achieves the best overall classification performance. This indicates that the performance improvement of the proposed model does not depend on a single module, but instead arises from the synergistic effect of node relationship modeling and local temporal dynamics modeling.

In terms of FNR and F1-score, compared with the ModernTCN backbone baseline model, incorporating the GCN+ module reduces the FNR from 0.1278 to 0.0602, corresponding to a reduction of 6.76 percentage points; the F1-score increases from 0.9317 to 0.9615, corresponding to an improvement of 2.98 percentage points. For the cable fire early warning task, the decrease in FNR indicates that the risk of missing fire samples is reduced, whereas the improvement in F1-score suggests that the model achieves a better overall balance between precision and recall. This result indicates that node relationship modeling helps the model utilize the collaborative response information among multiple sensors, thereby enhancing its ability to identify fire samples.

Similarly, in terms of FNR and F1-score, compared with the ModernTCN backbone baseline model, the classification performance of the model is further improved after incorporating the GRU module. The FNR decreases from 0.1278 to 0.0526, corresponding to a reduction of 7.52 percentage points; the F1-score increases from 0.9317 to 0.9692, corresponding to an improvement of 3.75 percentage points. This indicates that the introduction of the GRU module helps reduce the risk of missing fire samples and improves the overall balance between precision and recall. In addition, the AUC increases from 0.9609 to 0.9962, corresponding to an improvement of 3.53 percentage points, indicating that the model also achieves enhanced discrimination ability for fire samples under different decision thresholds. From the perspective of task characteristics, this improvement may be related to the complementary modeling of local dynamic information by the GRU module, enabling the model to better capture relatively weak but evolving temporal signals in the early stage of fire.

After the GCN+ and GRU modules are jointly incorporated into the ModernTCN temporal backbone, the complete proposed model achieves the best overall performance in terms of Accuracy, TPR, FNR, F1-score, and AUC, while maintaining a low FPR. In the ablation results, the standalone ModernTCN backbone obtains the lowest FPR of 0.0000, indicating a conservative tendency in identifying non-fire samples on the test set. However, its TPR and FNR are 0.8722 and 0.1278, respectively, showing that this conservative behavior is accompanied by a higher missed-detection risk for fire samples. After integrating GCN+ and GRU, the proposed model improves the TPR to 0.9850 and reduces the FNR to 0.0150, while the FPR remains low at 0.0080. These results indicate that the additional spatial-correlation and temporal-enhancement modules improve fire-sample detection capability while preserving a low false-alarm level.

Overall, the role of the GCN+ module is mainly reflected in enhancing the representation of collaborative relationships among multi-sensor nodes, whereas the role of the GRU module is mainly reflected in the complementary modeling of local temporal dynamics. After the two modules are jointly incorporated, the model can simultaneously utilize the temporal evolution features and node relationship information in early fire signals, thereby achieving better overall classification performance, stronger fire-sample identification capability, and a lower risk of missed detection.

## 4. Conclusions

Motivated by the need for early warning of cable fires in power cable tunnels, this study investigates the joint modeling of temporal evolution features and sensor-node spatial relationships using cable-fire records from a publicly available indoor EN 54 fire-test-room dataset. Considering the characteristics of weak early-stage fire signals, subtle local dynamic variations, and response differences among sensor nodes, this study constructed a multi-sensor spatiotemporal feature fusion model integrating GRU, ModernTCN, and GCN+. Comparative and ablation experiments were conducted to verify the effectiveness of the proposed model and the contributions of its components. The main conclusions are as follows.

(1) An early cable fire prediction task was formulated. In this study, multi-sensor time-series data collected over the 60 s preceding the current time *t* are used as the input to predict whether a cable fire occurs at the future time point *t* + 180 s. This formulation extends fire recognition from current-state discrimination to early warning oriented toward future risk. This task setting places greater emphasis on early variation information in sensor responses before fire occurrence. Under the binary prediction framework of this study, samples predicted as the fire state can be regarded as single-level early-warning decision outputs, thereby providing a model-level discriminative basis for early warning of cable fires in power cable tunnels.
(2) A multi-sensor spatiotemporal feature fusion model integrating GRU, ModernTCN, and GCN+ was proposed. The model performs short-term dynamic encoding of the input sequence through GRU, thereby enhancing its representation of local temporal dynamics during the early stage of fire development. ModernTCN is used to extract temporal evolution features from multivariate sensor sequences, while GCN+ is introduced to model spatial relationships among sensor nodes. On this basis, the model achieves a joint representation of short-term dynamics, temporal evolution, and node relationships, thereby improving its ability to represent weak anomalous signals during the early stage of fire development.
(3) The overall predictive advantage of the proposed model and the complementary effects of short-term dynamic encoding and node relationship modeling were verified. Comparative experimental results show that the proposed model achieves better overall predictive performance than typical temporal models, including LSTM, GRU, and TCN. On the test set, its accuracy, AUC, and F1-score reach 0.9884, 0.9994, and 0.9887, respectively, while the FNR decreases to 0.0150. These results indicate that the proposed model can effectively reduce the missed-detection risk for actual fire samples while maintaining high overall classification performance, making it more consistent with the engineering requirement for low missed-detection risk in early warning of cable fires in power cable tunnels. Ablation experiments further indicate that GRU and GCN+ provide complementary enhancements to the ModernTCN backbone. Specifically, GRU mainly improves the model’s ability to capture continuous weak variations, local fluctuations, and short-term dependency information during the early stage of fire development, whereas GCN+ enhances the model’s representational capacity for spatial correlations among sensor nodes and cross-node coordinated response patterns.
(4) This study still has certain limitations. The experimental data were obtained from a publicly available indoor EN 54 fire-test-room dataset, from which cable-fire-related sample segments were selected for controlled laboratory evaluation. However, real-world power cable tunnels may differ from the experimental environment in terms of spatial scale, ventilation conditions, cable layout, background disturbances, and sensor installation configurations. Therefore, the cross-scenario generalization capability of the model requires further validation. Future work may incorporate operational data from real-world power cable tunnels to conduct cross-scenario validation, analyze model performance under different prediction lead times, and further investigate graded early-warning strategies and online inference efficiency optimization, thereby improving the applicability of the model in practical engineering early-warning systems.

## Figures and Tables

**Table 1:** Sensor modules and measured variables of each multi-sensor node in the source dataset.

Sensor	Manufacturer	Measurand	Unit
SPS30	Sensirion AG, Stäfa, Switzerland	PM	cm^−3^
SVM40	Sensirion AG, Stäfa, Switzerland	VOC	A.U.
CO/MF-1000	MEMBRAPOR AG, Wallisellen, Switzerland	CO	ppm
UST6xxx	UST Umweltsensortechnik GmbH, Geratal, Germany	H_2_	ppm
SCD40	Sensirion AG, Stäfa, Switzerland	CO_2_	ppm
UVTRON	Hamamatsu Photonics K.K., Hamamatsu, Japan	UV photon	#
SGP40	Sensirion AG, Stäfa, Switzerland	Temperature, relative air humidity	°C, %

Note: PM, particulate matter; VOC, volatile organic compound; CO, carbon monoxide; H_2_, hydrogen; CO_2_, carbon dioxide; A.U., arbitrary unit; #, UV photon counts.

**Table 2:** Comparison results of different methods.

Model	Accuracy	TPR	FNR	FPR	F1-Score	AUC
LSTM	0.9612	0.9323	0.0677	**0.0080**	0.9612	0.9831
GRU	0.9574	0.9248	0.0752	**0.0080**	0.9572	0.9787
TCN	0.9535	0.9399	0.0602	0.0320	0.9542	0.9649
Ours	**0.9884**	**0.9850**	**0.0150**	**0.0080**	**0.9887**	**0.9994**

Note: Values are reported on the test set and rounded to four decimal places. Bold indicates the best or tied best result for each metric.

**Table 3:** Ablation study of the proposed model.

Model	Accuracy	TPR	FNR	FPR	F1-Score	AUC
ModernTCN	0.9341	0.8722	0.1278	**0.0000**	0.9317	0.9609
ModernTCN + GCN+	0.9612	0.9399	0.0602	0.0160	0.9615	0.9604
ModernTCN + GRU	0.9690	0.9474	0.0526	0.0080	0.9692	0.9962
Ours	**0.9884**	**0.9850**	**0.0150**	0.0080	**0.9887**	**0.9994**

## Data Availability

The original contributions presented in the study are included in the Article. Further inquiries can be directed to the corresponding author.
